# Association of Enamel Tooth Color of Central Incisors, Lateral Incisors, and Canines With Age and Gender to Aid in Forensic Identification: A Cross-Sectional Study

**DOI:** 10.7759/cureus.84865

**Published:** 2025-05-26

**Authors:** Madhuri S Sale, Chetan Patil, Tanushree Dalvi, Mahesh R Khairnar, Amol Karagir, Anil Patil

**Affiliations:** 1 Oral Pathology and Microbiology, Bharati Vidyapeeth (Deemed to be University) Dental College and Hospital, Sangli, IND; 2 Public Health Dentistry, Bharati Vidyapeeth (Deemed to be University) Dental College and Hospital, Sangli, IND; 3 Public Health Dentistry, Faculty of Dental Sciences, Institute of Medical Sciences, Banaras Hindu University, Varanasi, IND; 4 Oral Medicine and Radiology, Bharati Vidyapeeth (Deemed to be University) Dental College and Hospital, Sangli, IND; 5 Pediatric and Preventive Dentistry, Bharati Vidyapeeth (Deemed to be University) Dental College and Hospital, Sangli, IND

**Keywords:** cross-sectional study, enamel color, forensic odontology, maxillary incisors and canine, shade guide

## Abstract

Aims and objectives

Forensic dentistry plays a crucial role in human identification, and tooth enamel color can serve as a valuable tool in forensic investigations. This cross-sectional study assesses the enamel shade of maxillary central and lateral incisors and canines among different age groups and genders, as well as dietary preferences, to establish patterns that can assist forensic identification.

Materials and methods

A total of 370 participants were recruited through random sampling at Bharati Vidyapeeth Dental College and Hospital, Sangli. The VITA classical shade guide (VITA Zahnfabrik, Bad Säckingen, Germany) was used to assess the enamel color under natural daylight conditions.

Results

Statistical analysis revealed significant differences in enamel shades between males and females but no significant differences based on diet.

Conclusions

The findings highlight the relevance of enamel shade analysis in forensic identification and emphasize the need for further research to strengthen its applicability in forensic sciences.

## Introduction

Forensic science includes a specialized field, forensic odontology, which utilizes dental records for human identification. Various extrinsic and intrinsic factors, like age, gender, and dietary habits, contribute to enamel color variations. Enamel color, a unique dental feature influenced by genetic and environmental factors, can serve as a distinguishing trait in forensic investigations. Identification of deceased victims in forensic odontology is important for age estimation, unknown individuals, and also for crimes and accidents [1,2]. Enamel shades are particularly useful in forensic cases involving unidentifiable bodies or missing persons. Teeth can be used to estimate age in forensic science as they are resistant to deterioration and decay [3,4]. The stability of tooth morphological traits makes them a popular tool to determine age with accuracy [3]. Because of this, teeth can be used as a helpful indicator of age. In recent years, tooth color matching has attracted attention in the area of dentistry. Many authors have suggested that with age, the color of all teeth becomes dark [5]. Furthermore, a deceased individual’s age estimation can be significant in forensic odontology.

The enamel color of teeth changes in proportion with age and is influenced by both external and internal tooth anatomy [6,7]. Time‑consuming techniques for age estimation may be replaced if a time-effective and valid alternative is available [5]. Many parameters, like morphological and radiological parameters, cementum and thickness of dentin, Gustafson's method, volume of pulp-tooth ratio, and incremental lines, can be used to determine age. Still, these methods are time-consuming and may be eliminated if a reliable and efficient substitute is noted [6].

Earlier research studies have explored variations in enamel shade, but limited research has focused on gender-based and dietary influences on tooth color. Very few studies using enamel shade for age estimation have been reported; hence, keeping this in mind, this study was planned to evaluate the relationship between enamel shade and chronological age. This study aimed to assess the enamel shade of maxillary incisors and canines among different genders and dietary groups, providing insights that can aid forensic identification.

## Materials and methods

Sample selection and study design

The present cross-sectional study was conducted at a dental institution in Sangli after obtaining ethical approval from the Institutional Ethics Committee of Bharati Vidyapeeth (Deemed to be University) Medical College & Hospital, Sangli (approval no. BV (DU) MC&H/Sangli/IEC/D-97/23). A total of 370 participants were included using a random sampling method, with prior written and informed consent from each participant. Participants aged 18 years and above who visited the dental outpatient department (OPD) were recruited in this study. Individuals with traumatized teeth, dental fluorosis, a history of tooth bleaching, tobacco consumption, dental caries, occupational habits affecting enamel color, or birth trauma were excluded from this study.

Sample size calculation

The sample size was calculated using the Raosoft Sample Size Calculator (Raosoft, Inc., Seattle, USA). The following parameters were considered for sample size estimation: population size: 3000 (Average OPD for three months); Margin of error (MOE): 5%; Confidence level: 95%; and response distribution/sample proportion: 50%. 

Using the formula $n = \frac{N \cdot X}{X + N - 1}$, where $X = \frac{Z_{\alpha/2}^2 \cdot p \cdot (1 - p)}{\text{MOE}^2}$ and $Z_{\alpha/2}$ is the critical value of the normal distribution at $\alpha/2$ (e.g., for a confidence level of 95%, $\alpha = 0.05$ and the critical value is 1.96), MOE is the margin of error, $p$ is the sample proportion, and $N$ is the population size. The sample size calculated using the above formula was 341.

Calibration and reliability of shade selection

Two trained and calibrated examiners (MSS and CP) evaluated the tooth shades. Calibration was done on a set of 30 individuals who were not part of the main study. Any doubts or confusions regarding shade selection were resolved through discussion and by consulting a third examiner (AK), if needed. The reliability of the shade selection was analyzed using kappa statistics. The kappa statistics showed an almost perfect agreement (k = 0.856) between the two examiners in terms of the shade selection.

Shade Assessment Procedure

**Figure 1:**
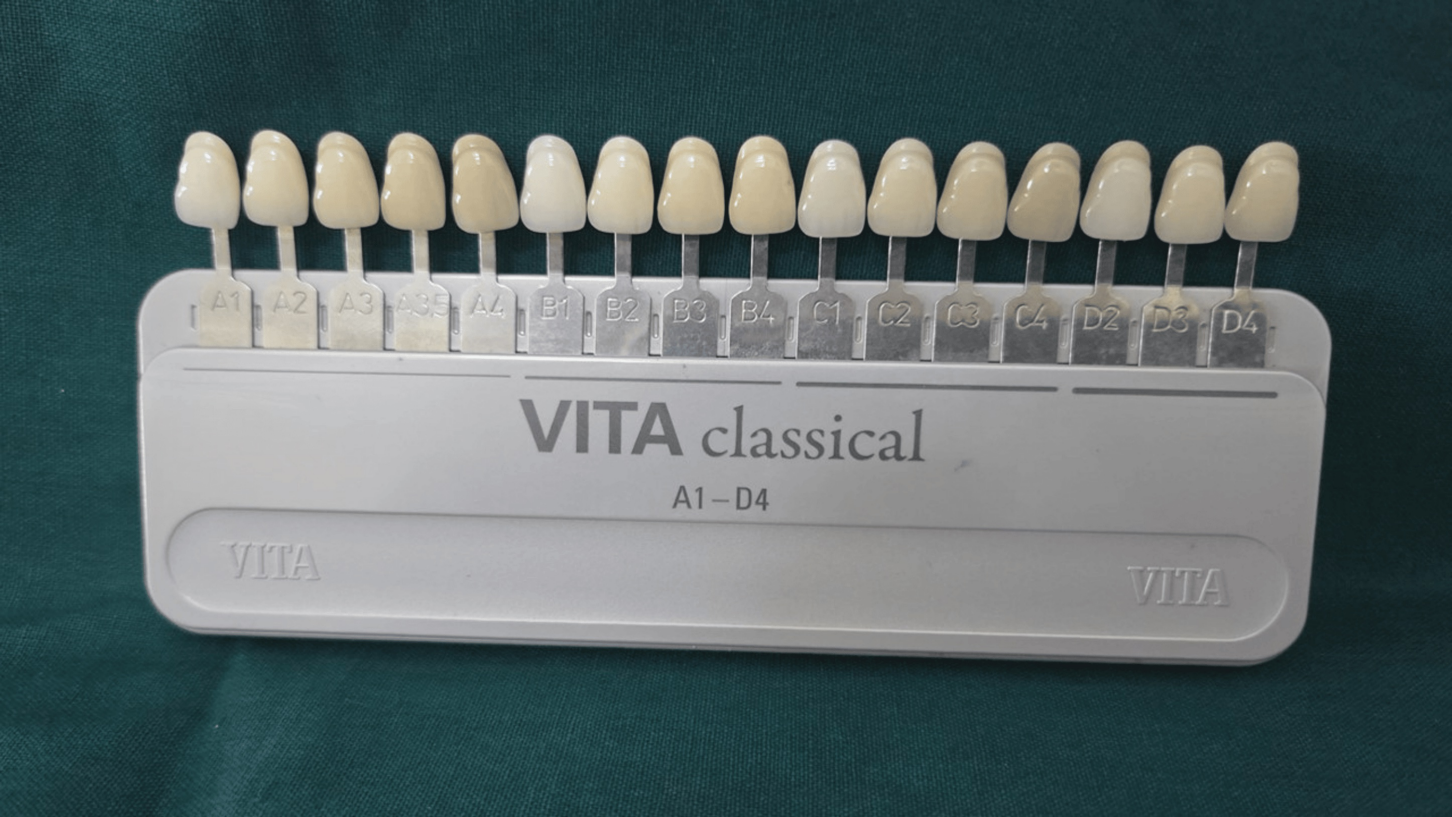
VITA classical shade guide: the shades ranges from A1-A4, B1-B4, C1-C4, and D2-D4 VITA classical shade guide by VITA Zahnfabrik, Bad Säckingen, Germany.

VITA classical shade guide (VITA Zahnfabrik, Bad Säckingen, Germany) was utilized to assess enamel color (Figure 1).

**Figure 2:**
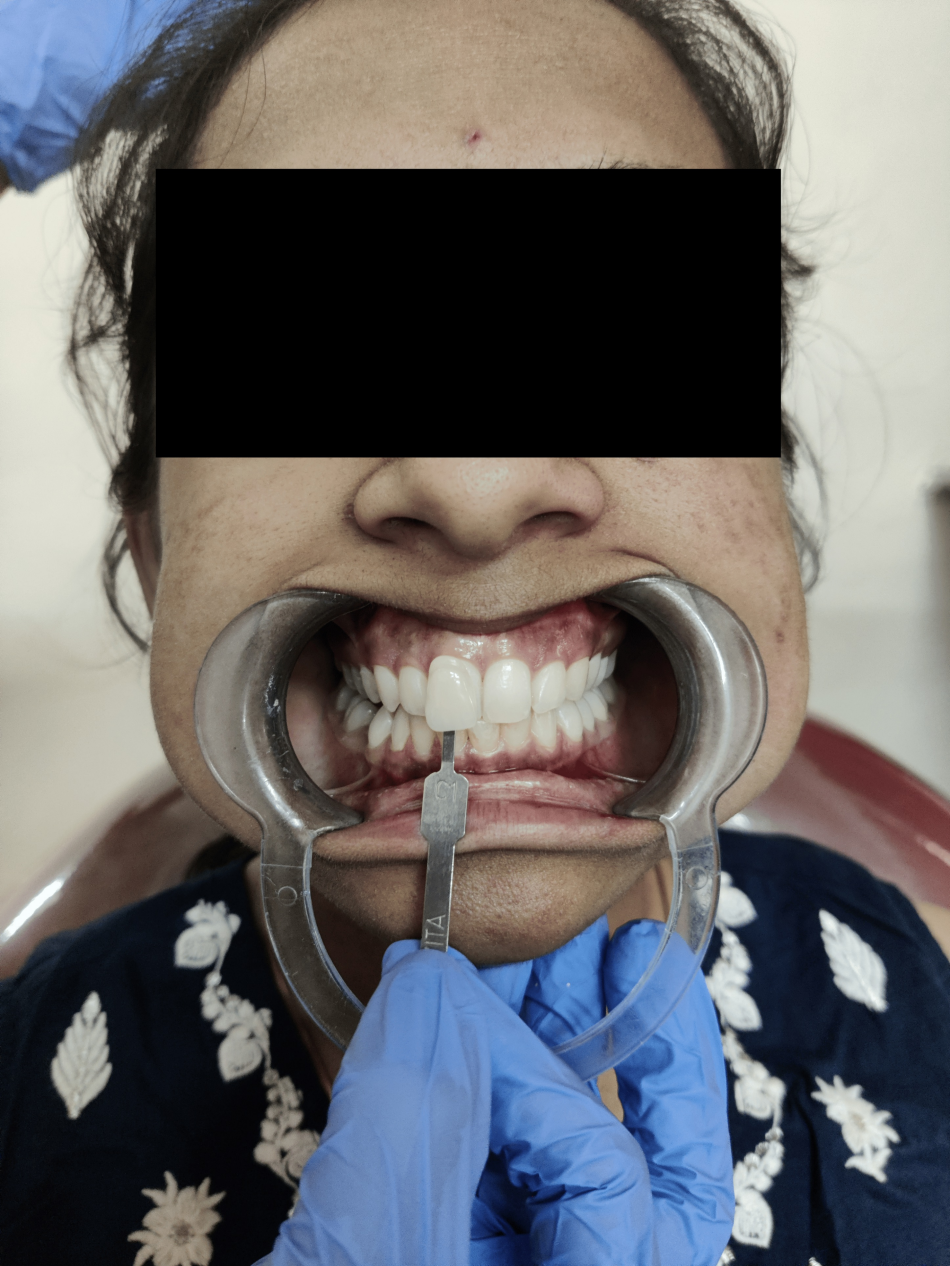
Shade matching under natural light using VITA classical shade guide VITA classical shade guide by VITA Zahnfabrik, Bad Säckingen, Germany.

On the basis of hue difference, the VITA classical shade guide was arranged from A to D. The shade ranges were A1-A4, B1-B4, C1-C4, and D2-D4. As the number increased, the value decreased, whereas the chroma increased simultaneously [8]. Oral prophylaxis was performed prior to the shade identification procedure. Assessment of enamel shade was conducted under natural sunlight while the participants were seated in a dental chair. The maxillary central incisor, lateral incisor, and canine were used for shade matching, and the teeth were viewed at eye level (Figure 2).

The middle third of the labial surface and the shade guide were matched. The shade guide was held parallel to the selected tooth at an arm’s length. Each tooth was observed for 4 to 5 seconds. First, the value of tooth shade was assessed, followed by chroma and hue. The findings were evaluated by an observer to ensure accuracy. 

Statistical analysis

Data was collected and entered in Excel (Microsoft Corp., Redmond, USA) and analyzed statistically using SPSS software (IBM Corp., Armonk, USA) to identify significant differences in enamel shade distribution based on gender and dietary habits. Chi-squared test was utilized to determine the level of significance among different variables. The level of significance was set at 5%.

## Results

The study analyzed enamel shade variations among males and females for the maxillary central incisors, lateral incisors, and canines among 370 participants. Among 370 participants, 217 (58.7%) were females and 153 (41.4%) were males. 27.74 years was the mean age of study participants (Table 1).

**Table 1:** Demographic details of study participants

Variable	Value
Age (in years)	27.74 (SD=11.16)
Gender, n (%)	Female: 217 (58.6%) Male: 153 (41.4%)

Table 2 shows enamel shade comparison in the central incisor, lateral incisor, and canine among male and female participants as analysed using the chi-squared test. The most common shades of central incisors among males and females were B1 (46.50%) and B2 (34%), respectively, and there was a significant difference in enamel shades of central incisors among males and females (p ≤ 0.001). Similarly, the most common shades of lateral incisors among males and females were B1 (41.50%) and B2 (32.70%), respectively, and there was a significant difference in the enamel shades of lateral incisors among males and females (p ≤ 0.004). The most common shades of canines among males and females were B3 (39.20%) and B4 (31.40%), respectively, and there was a significant difference in the enamel shades of central incisors among males and females (p ≤ 0.001).

**Table 2:** Comparison of enamel shade among male and female subjects F: Female; M: Male; *Significant

Shade		Central Incisor		Lateral Incisor		Canine	
		F	M	F	M	F	M
A1	n	43	39	32	35	3	0
	%	19.80%	25.50%	14.70%	22.90%	1.40%	0.00%
A2	n	21	16	29	19	37	39
	%	9.70%	10.50%	13.40%	12.40%	17.10%	25.50%
A3	n	1	3	4	4	21	19
	%	0.50%	2.00%	1.80%	2.60%	9.70%	12.40%
B1	n	101	32	90	30	1	0
	%	46.50%	20.90%	41.50%	19.60%	0.50%	0.00%
B2	n	33	52	42	50	17	0
	%	15.20%	34.00%	19.40%	32.70%	7.80%	0.00%
B3	n	7	3	9	7	85	34
	%	3.20%	2.00%	4.10%	4.60%	39.20%	22.20%
B4	n	2	0	1	0	35	48
	%	0.90%	0.00%	0.50%	0.00%	16.10%	31.40%
C1	n	4	3	1	2	9	5
	%	1.80%	2.00%	0.50%	1.30%	4.10%	3.30%
C2	n	5	4	7	5	5	4
	%	2.30%	2.60%	3.20%	3.30%	2.30%	2.60%
C3	n	0	0	1	0	4	3
	%	0.00%	0.00%	0.50%	0.00%	1.80%	2.00%
D1	n	0	0	1	0	0	0
	%	0.00%	0.00%	0.50%	0.00%	0.00%	0.00%
D2	n	0	1	0	1	0	0
	%	0.00%	0.70%	0.00%	0.70%	0.00%	0.00%
D3	n	0	0	0	0	0	1
	%	0.00%	0.00%	0.00%	0.00%	0.00%	0.70%
p-value		<0.001*		0.004*		<0.001*	

The enamel shades of central incisors, lateral incisors, and canines were also compared among vegetarian and mixed-diet individuals using the chi-squared test. Table 3 shows enamel shade comparison in the central incisors, lateral incisors, and canines according to diet. The most common shade of central incisors among vegetarian and mixed diet subjects was B1 (34.20 and 36.80%, respectively), and there was a non-significant difference in the enamel shades of central incisors among vegetarian and mixed diet subjects (p = 0.221). The most common shade of lateral incisors among vegetarian and mixed diet subjects was B1 (28.30 and 34.40%, respectively), and there was a non-significant difference in the enamel shades of central incisors among vegetarian and mixed diet subjects (p = 0.595). The most common shade of canines among vegetarian and mixed diet subjects was B2 (30.80% and 32.80%, respectively), and there was a non-significant difference in the enamel shades of canines among vegetarian and mixed diet subjects (p = 0.83). Both vegetarians and mixed-diet individuals displayed similar enamel shade patterns across all assessed tooth types. 

**Table 3:** Comparison of enamel shade according to diet

Shade		Central Incisor		Lateral Incisor		Canine	
		Veg	Mixed	Veg	Mixed	Veg	Mixed
A1	n	27	55	19	48	1	2
	%	22.50%	22.00%	15.80%	19.20%	0.80%	0.80%
A2	n	14	23	20	28	27	49
	%	11.70%	9.20%	16.70%	11.20%	22.50%	19.60%
A3	n	1	3	3	5	14	26
	%	0.80%	1.20%	2.50%	2.00%	11.70%	10.40%
A4	n	0	0	0	0	0	1
	%	0.00%	0.00%	0.00%	0.00%	0.00%	0.40%
B1	n	41	92	34	86	5	12
	%	34.20%	36.80%	28.30%	34.40%	4.20%	4.80%
B2	n	22	63	29	63	37	82
	%	18.30%	25.20%	24.20%	25.20%	30.80%	32.80%
B3	n	7	3	8	8	22	61
	%	5.80%	1.20%	6.70%	3.20%	18.30%	24.40%
B4	n	1	1	0	1	7	7
	%	0.80%	0.40%	0.00%	0.40%	5.80%	2.80%
C1	n	2	5	1	2	0	0
	%	1.70%	2.00%	0.80%	0.80%	0.00%	0.00%
C2	n	5	4	6	6	4	5
	%	4.20%	1.60%	5.00%	2.40%	3.30%	2.00%
C3	n	0	0	0	1	3	4
	%	0.00%	0.00%	0.00%	0.40%	2.50%	1.60%
D1	n	0	0	0	1	0	0
	%	0.00%	0.00%	0.00%	0.40%	0.00%	0.00%
D2	n	0	1	0	1	0	0
	%	0.00%	0.40%	0.00%	0.40%	0.00%	0.00%
D3	n	0	0	0	0	0	1
	%	0.00%	0.00%	0.00%	0.00%	0.00%	0.40%
p-value		0.221		0.595		0.83	

## Discussion

The present study evaluated the association between enamel tooth color of central incisors, lateral incisors, and canines with age and gender to aid in forensic identification among 370 participants using the VITA classic shade guide. The reason for selecting the VITA classic shade guide was that it is durable and cost-effective. It does not require frequent replacement of the shade guide. It provides an efficient comparison with natural tooth color. To communicate the proper tooth color, brightness, and translucence, this technique is used by dentists, dental laboratory technicians, and dental assistants [8,9]. However, there are some limitations to shade guide usage. This technique is influenced by age, gender, observer skill, eye fatigue, and surrounding light [10].

Gender differences in enamel shade

The individual's sex, race, and heredity have an impact on tooth color [11]. The skin and tooth colors of various ethnic groups significantly correlate, supporting the idea that genetics influences tooth color change. The thickness, translucency, and chemical makeup of enamel and dentin combine to create enamel shade. The study revealed significant gender-based differences in enamel shades. Males exhibited lighter shades in incisors (B1), whereas females showed a slightly darker shade (B2), which was in accordance with the study done by Patel et al. [5]. This could be attributed to differences in enamel thickness, mineral composition, and genetic factors influencing tooth color. Canine teeth in males were found to have a darker shade (B3), while in females, an even darker shade (B4) was observed. In contrast, Vaidya et al. and Singh et al. found that in none of the age groups was there any statistically significant difference observed in the color of enamel in males and females (p < 0.05) [7,12]. Canines undergo higher functional stress, and variations in wear patterns may contribute to these differences. Due to changes in dentin and enamel thickness, the teeth's shade varies from the incisal to the gingival region. The middle third of the tooth is the optimum place to measure the shade [13].

These findings align with previous research suggesting that males generally have lighter enamel due to higher enamel translucency, whereas females may exhibit darker shades due to variations in dentin thickness and hormonal influences. However, visual shade selection varies, depending on the clinician's color perception and experience, ambient light conditions, background of the tooth, and the shade guide used [14].

Influence of diet on enamel shade

No significant differences were found between vegetarians and mixed-diet individuals regarding enamel shade. While diet influences extrinsic staining, it appears to have minimal impact on inherent enamel color. Previous research done by De Geus et al. and Jamwal et al. suggests that dietary habits rich in colored foods and beverages contribute to external staining, but intrinsic enamel shade remains largely unaffected [15,16]. Another meta-analysis, done by Smits et al., revealed that people having a vegetarian diet are at a greater risk of erosion, which may affect the enamel color [17].

Strengths and limitations

The present research has followed the Strengthening the Reporting of Observational Studies in Epidemiology (STROBE) guidelines, and the sample size was scientifically calculated, which improved the credibility of the study. The study used a visual method of color estimation using the VITA classical shade guide, which has a few limitations - it is subjective, and the color of the shade guide may vary according to the brand.

Forensic implications

Enamel shade assessment can serve as a valuable forensic tool for human identification. The significant gender-based differences in enamel shades can aid in sex determination during forensic investigations. Additionally, tooth color analysis can complement other forensic methods, such as dental charting and DNA analysis, in identifying unknown individuals. The study also highlights the stability of enamel color across different diets, reinforcing its reliability as a forensic identifier. Further research incorporating larger sample sizes and additional variables such as age and ethnicity can strengthen the application of enamel shade analysis in forensic casework.

## Conclusions

This study provides valuable insights into enamel shade variations among different genders and dietary groups. The findings show notable distinctions in enamel shades between males and females, with males exhibiting lighter shades in incisors and darker shades in canines compared to females. However, diet was found to have no significant impact on enamel color.

The findings highlight the potential forensic application of enamel shade analysis in sex determination and human identification. Future studies should explore additional factors such as aging effects and genetic influences to further establish enamel shade assessment as a reliable forensic tool.
